# Severe Klippel-Feil syndrome with Mondini malformation of inner ear

**DOI:** 10.11604/pamj.2014.19.254.4998

**Published:** 2014-11-07

**Authors:** Aqeel Abdullah Alaqeel

**Affiliations:** 1 Children Hospital, King Fahad Medical City, Riyadh, Saudi Arabia

**Keywords:** Klippel-Feil syndrome, Mondini malformation, congenital

## Abstract

Klippel-Feil syndrome is defined as the fusion of cervical vertebra with associated congenital anomalies but was rarely reported to be associated with Mondini Malformation. We report a newborn girl with severe neck extension, computed tomography (CT) of the neck after birth showed fusion of the fifth, sixth, and seventh cervical vertebrae, compatible with Klippel-Feil Syndrome and CT temporal bone showed choclear dysplasia with incomplete number of turns that is compatible with Mondini Malformation.

## Introduction

Klippel-Feil Syndrome is a rare congenital disorder characterized by fusion of two cervical vertebrae or more at any level. Klippel-Feil Syndrome uncommonly has been reported with inner ear malformation. Mondini Malformation is a complex malformation in which the normal cochlear two and a half turn spirals were replaced by a hypoplastic coil of one and a half turns because of an absence of the interscalar septum [1].

## Patient and observation

We report a unique case of a neonate delivered by elective cesarean section to a 38-year-old mother G8 P6 +1. Antenatal scan showed severe neck extension, and was suspected to have Cystic Hygroma. Birth weight was 3470 grams. Apgars were 6 at 1 minute and 8 at 5 minutes respectively. The head appeared to be placed directly on the trunk posteriorly between the shoulders, with restricted neck movements. Baby was of short stature (42 cm) with a short and webbed neck. A low hair line was noted. Scapulae were higher than normal and no dysmorphic features. The Baby was immediately intubated after birth and mechanically ventilated to secure the airway and was extubated to nasal cannula with acceptable oxygen saturation at 5 days of age.

**Figure 1:**
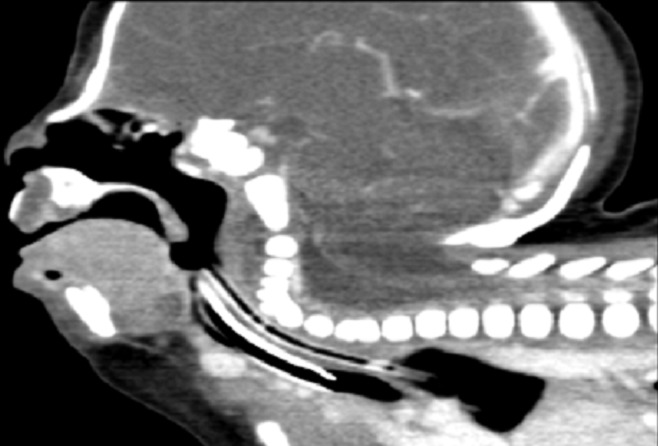
Severe hyperextension of the cervical spine. Fusion of 5^th^, 6^th^, & 7^th^ cervical vertebrae. Widened foramen magnum and spinal canal

**Figure 2:**
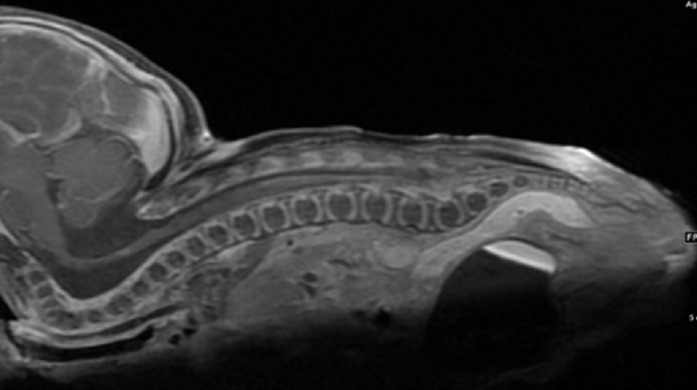
Fused vertebral bodies and absent posterior elements seen throughout the cervical spine with severe hyperextension. The cervical spine is flattened and there is splitting of the cervical portion of the cord extending up into the lower medulla

**Figure 3:**
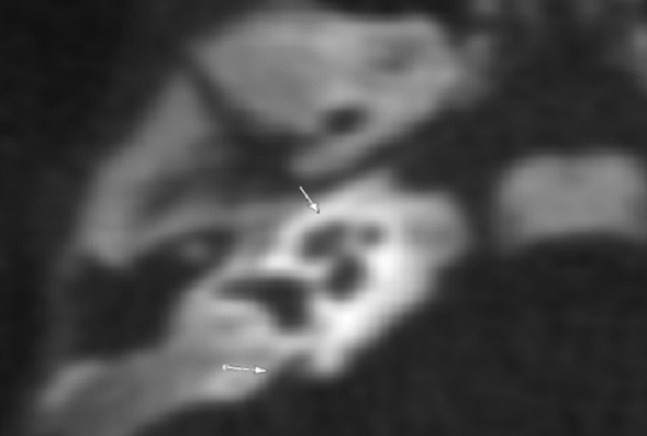
CT scan of temporal bone shows: Upper arrow: incomplete number of turns. Lower arrow: Widened vestibular aqueducts

Several investigations were ordered for the baby including a chromosomal analysis which was normal, cardiovascular imaging which showed atrial septal defect with small patent ductus arteriousus, and Renal and abdominal ultrasound which were unremarkable, CT neck which showed fusion of the fifth, sixth, and seventh cervical vertebrae (Figure 1), MRI spine which showed fused vertebral bodies and absent posterior elements seen throughout the cervical spine with severe hyperextension, with flattened cervical spine and splitting of the cervical portion of the cord extending up into the lower medulla (Figure 2), and CT temporal bone showed vestibular aqueduct on both sides, choclear dysplasia with incomplete number of turns that is compatible with Mondini Malformation (Figure 3).

During hospitalization the baby had frequent apneas with nasal cannula and eventually required a tracheostomy tube at 2 months of age for optimal oxygenation, after which the patient became asymptomatic. Infant hearing screening was performed on multiple settings which revealed failed hearing test for both ears that was compatible with the radiological finding, and he was planned for cochlear implantation at 6 months of age. No operation was done to correct his underlying spinal deformity.

## Discussion

Klippel-Feil Syndrome consists of congenital fusion of cervical vertebrae Associated with other anomalies including renal, heart, central nervous system, spinal and limb defects. The classic clinical triad includes a short neck, limb defects, low hairline and limitation in movement of the neck. The triad is seen in only 40% to 50% of Klippel-Feil Syndrome patients. [1–3] It is a result of failure of the normal segmentation of the cervical somites during the 3rd to 8th weeks of gestation.[5] It was first reported in 1912 and divided to 3 types [4, 5]: type I is fusion of many of the cervical and upper thoracic vertebrae, type 2 is fusion at one or two interspaces with occipitoatlantoid fusion, hemivertebrae or other abnormalities in the cervical spine, and type III is cervical fusion in combination with lower thoracic or lumbar fusion [5, 6]. The morphologic features described in our patient were consistent with type II Klippel-Feil Syndrome

Deafness can be part of Klippel-Feil Syndrome and may be conductive, sensorineural or a mixed deafness. McGaughran et al reported the audiological assessment of forty four patients with Klippel-Feil Syndrome, thirty five were found to have abnormalities on audiological testing. [3] Mondini dysplasia has been associated with different syndromes incluidng Pendred Syndrome, CHARGE Syndrome, Klippel-Feil Syndrome,DiGeorge Syndrome, and Wildervanck Syndrome [7]. Up to author′s knowledge, Klippel-Feil Syndrome was rarely reported to be associated with Mondini Malformation; it was reported by Yang et al in 1997 in 3 cases with sensorineural hearing loss cases that were previously considered to be of unknown cause [8]. Klippel-Feil Syndrome is one of the congenital anomalies that cause difficult airway which requires precaution while performing intubation due to the associated short neck, limited neck movement and cervical instability [7], in our case, the neck extension was severe enough to require immediate intubation after delivery.

## Conclusion

Klippel-Feil Syndrome is associated with multiple congenital anomalies, therefore hearing screening is mandatory and inner ear imaging is necessary if the test is negative to rule out inner ear malformation including Mandoni malformation.
